# Pms2 Suppresses Large Expansions of the (GAA·TTC)_n_ Sequence in Neuronal Tissues

**DOI:** 10.1371/journal.pone.0047085

**Published:** 2012-10-11

**Authors:** Rebecka L. Bourn, Irene De Biase, Ricardo Mouro Pinto, Chiranjeevi Sandi, Sahar Al-Mahdawi, Mark A. Pook, Sanjay I. Bidichandani

**Affiliations:** 1 Department of Biochemistry and Molecular Biology, University of Oklahoma Health Sciences Center, Oklahoma City, Oklahoma, United States of America; 2 Department of Pediatrics, University of Oklahoma Health Sciences Center, Oklahoma City, Oklahoma, United States of America; 3 Biosciences Division, School of Health Sciences and Social Care, Brunel University, Uxbridge, United Kingdom; University of Minnesota, United States of America

## Abstract

Expanded trinucleotide repeat sequences are the cause of several inherited neurodegenerative diseases. Disease pathogenesis is correlated with several features of somatic instability of these sequences, including further large expansions in postmitotic tissues. The presence of somatic expansions in postmitotic tissues is consistent with DNA repair being a major determinant of somatic instability. Indeed, proteins in the mismatch repair (MMR) pathway are required for instability of the expanded (CAG·CTG)_n_ sequence, likely via recognition of intrastrand hairpins by MutSβ. It is not clear if or how MMR would affect instability of disease-causing expanded trinucleotide repeat sequences that adopt secondary structures other than hairpins, such as the triplex/R-loop forming (GAA·TTC)_n_ sequence that causes Friedreich ataxia. We analyzed somatic instability in transgenic mice that carry an expanded (GAA·TTC)_n_ sequence in the context of the human *FXN* locus and lack the individual MMR proteins Msh2, Msh6 or Pms2. The absence of Msh2 or Msh6 resulted in a dramatic reduction in somatic mutations, indicating that mammalian MMR promotes instability of the (GAA·TTC)_n_ sequence via MutSα. The absence of Pms2 resulted in increased accumulation of large expansions in the nervous system (cerebellum, cerebrum, and dorsal root ganglia) but not in non-neuronal tissues (heart and kidney), without affecting the prevalence of contractions. Pms2 suppressed large expansions specifically in tissues showing MutSα-dependent somatic instability, suggesting that they may act on the same lesion or structure associated with the expanded (GAA·TTC)_n_ sequence. We conclude that Pms2 specifically suppresses large expansions of a pathogenic trinucleotide repeat sequence in neuronal tissues, possibly acting independently of the canonical MMR pathway.

## Introduction

Expanded trinucleotide repeat sequences are known to cause several inherited human neurodegenerative diseases [1]. These sequences are subject to changes in repeat length during parental transmission, a phenomenon termed intergenerational instability. In addition, expanded trinucleotide repeats undergo further large expansions in specific somatic tissues. Although both forms of instability appear to influence disease onset and progression, it is likely that intergenerational and somatic instability occur by distinct mechanisms. This report focuses specifically on uncovering the processes that influence somatic instability.

Several observations indicate that somatic instability resulting in progressive expansion within specific tissues contributes to disease pathogenesis and progression. First, the tissue-specificity of somatic instability tends to correlate with disease pathology. For example, in Huntington disease and myotonic dystrophy the expanded (CAG·CTG)_n_ sequence undergoes further large expansions in the striatum and muscle, respectively [2], [3]. Similarly, in Friedreich ataxia (FRDA), the pathogenic expanded (GAA·TTC)_n_ sequence in intron 1 of the *FXN* gene [4] undergoes progressive expansion in the cerebellum and dorsal root ganglia of FRDA patients [5] and in a transgenic mouse model [6]. Second, somatic instability is known to precede the development of the disease phenotype in a mouse model of Huntington disease [2], [3], [7]. Finally, the absence of somatic instability can ameliorate the disease phenotype. Genetic suppression of somatic instability in a mouse model for Huntington disease significantly delayed intracellular accumulation of mutant huntingtin protein, the onset of disease, and death [8]. Likewise, somatic instability seems to be important in the pathology of FRDA. In transgenic mice, an expanded (GAA·TTC)_n_ sequence within a human *FXN* transgene recapitulated the age-dependent and tissue-specific somatic instability observed in FRDA patients [6], and rescuing the embryonic-lethal *Fxn*-deficient mice with this transgene resulted in an FRDA-like phenotype [9], [10]. However, when a similar-sized (GAA·TTC)_n_ repeat was knocked-in into the mouse *Fxn* gene, the sequence did not show somatic instability, and it was not possible to replicate the FRDA phenotype [11]. Furthermore, an expanded (GAA·TTC)_n_ sequence in an intron of the At4g13430 gene was found to cause a growth defect in *Arabidopsis thaliana*, and this repeat also displayed somatic instability [12]. Therefore, understanding the molecular mechanisms of somatic instability of disease-causing expanded trinucleotide repeats in somatic tissues is of great interest.

The post-mitotic nature of tissues affected in trinucleotide expansion disorders indicates that DNA repair may be an important determinant of somatic instability. The highly conserved mismatch repair (MMR) system prevents point mutations, insertions and deletions. In *Escherichia coli*, MMR is initiated when a MutS homodimer recognizes and binds a mismatch or insertion-deletion loop (IDL) [13]. Mismatch binding stimulates ATP hydrolysis by MutS, which is required for progression of MMR. A MutL homodimer is recruited, and the MutS-MutL complex activates the latent endonuclease activity of MutH. The bases from the nick to the mispaired base(s) are excised and re-synthesized to repair the lesion. Mammalian MMR is initiated by a heterodimer of the MutS homologs Msh2, Msh3 and Msh6 [14]. MutSα (Msh2-Msh6) preferentially recognizes base-base mispairs and IDLs of one to two bases, while MutSβ (Msh2-Msh3) preferentially recognizes IDLs of two to ten bases. The mammalian MutL homologs Mlh1, Mlh3 and Pms2 also function as heterodimers. The primary MutL heterodimer, MutLα (Mlh1-Pms2), can be recruited by either MutSα or MutSβ. MutLγ (Mlh1-Mlh3) may be recruited by MutSα, but the role of MutLγ in MMR is unclear. There is no known mammalian MutH homolog, although there is evidence to suggest that Pms2 can function as an endonuclease [15], [16].

MMR deficiency causes microsatellite instability, a form of genomic instability characterized by widespread somatic length variability in short tandem repeats, typically involving few repeat units. Consistent with this role, MMR is known to prevent small length changes of expanded trinucleotide repeats [17]. However, proteins in the MMR pathway have been shown to promote large length changes of expanded (CAG·CTG)_n_ sequences. In mice, the absence of Msh2 or Msh3 was shown to eliminate somatic expansions of (CAG·CTG)_n_ [7], [18]–[22]. The absence of Msh6 did not reduce the frequency of somatic expansions in neuronal tissues, although it may affect instability in other tissues [20], [22]. These results indicate that MutSβ is required to produce somatic expansions of the expanded (CAG·CTG)_n_ sequence, and multiple lines of evidence suggest that somatic expansions occur through the MMR pathway itself. First, MutSβ promotes repair on CAG/CTG hairpin templates *in vitro* [23]. In addition, in mice lacking Pms2, the rate of somatic expansions of the expanded (CAG·CTG)_n_ sequence was reduced by ∼50% [24]. Finally, expansions were eliminated in mice homozygous for an ATPase-deficient mutation of *Msh2* [25]. These data indicate involvement of the downstream steps of MMR and support the role of an intact, but error-prone, MMR pathway in producing somatic expansions of the (CAG·CTG)_n_ sequence. However, it has also been proposed that MutSβ could produce expansions by acting independently of the other MMR proteins to create CAG/CTG hairpins or prevent their repair [26]–[28]. Indeed, many MMR proteins are known to have functions outside of the canonical MMR pathway; the mammalian MutS and MutL homologs are known to play important roles in processes such as class switch recombination, homologous recombination and DNA damage signaling [13]–[17]. Thus, it is possible that MMR proteins could act through various DNA modifying pathways to influence somatic instability.

The accuracy and efficiency of DNA repair, and which repair pathway(s) are activated, are in turn affected by the DNA sequence and structure [29]–[32]. Therefore, the effect of MMR proteins on trinucleotide repeat instability could vary depending on the sequence of the repeat motif and its secondary structure(s). While the (CAG·CTG)_n_ sequence forms intrastrand hairpins [33], [34], the (GAA·TTC)_n_ sequence tends to form triplex-based structures [35]–[40]. In yeast, MutSβ and MutLα promote fragility of expanded (GAA·TTC)_n_ sequences, leading to large deletions within the repeat tract [41]. However, the role of mammalian MMR proteins in tissue-specific expansions of the (GAA·TTC)_n_ sequence remains unclear.

To determine if and how MMR proteins affect instability of the (GAA·TTC)_n_ sequence in the context of mammalian tissues, we crossed the previously described YG8 transgenic mouse model [6], [42] with mice lacking the individual MMR proteins Msh2, Msh6, or Pms2 [43]–[45] and analyzed somatic instability by small-pool PCR [46]. Absence of Msh2 or Msh6 significantly stabilized the repeat tract. Remarkably, Pms2 deficiency increased the frequency of large expansions in the cerebellum, cerebrum, and dorsal root ganglia, but not in heart or kidney. The absence of Pms2 had no significant effect on contractions. Our results show that while mammalian MMR promotes somatic instability of the (GAA·TTC)_n_ sequence, Pms2 plays a unique role in suppressing large somatic expansions in neuronal tissues.

## Materials and Methods

### Transgenic mice, tissues and DNA samples

YG8 *FXN* GAA repeat expansion-containing transgenic mice were crossed with *Msh2*, *Msh6* or *Pms2* heterozygous knockout mice to establish double genetically modified mice as previously described [47]. Genomic DNA was isolated from multiple tissues of MMR-deficient and MMR-proficient YG8 mice using the DNeasy Blood & Tissue Kit (Qiagen). We previously showed that the YG8 mice developed substantial somatic instability by 10 to 12 months of age, and that instability was most marked in the cerebellum [6]. In order to test the effect of MMR we therefore collected tissues from mice at 10 to 12 months of age. Cerebellum was analyzed for all genotypes, and other selected tissues were analyzed as follows: 10-month old YG8-*Msh2* ^+/+^ and YG8-*Msh2* ^−/−^ (cerebellum, cerebrum, DRG, heart); 12-month old YG8-*Pms2* ^+/+^ and YG8-*Pms2* ^−/−^ (cerebellum, cerebrum, DRG, heart, kidney); 5-month old YG8-*Pms2* ^+/+^ and YG8-*Pms2* ^−/−^ (cerebellum); 11- or 12-month old YG8-*Msh6* ^+/+^ and YG8-*Msh6* ^−/−^ (cerebellum). The use of animals for this research project was reviewed and approved by the Institutional Animal Care and Use Committee at the University of Oklahoma Health Sciences Center (protocol 08-129), and the Animal Ethics Committee at Brunel University.

### Small-pool PCR analysis

Small-pool PCR was performed as previously described [46], [48]. Briefly, serial dilutions of DNA were made to obtain “small pools” of DNA containing 1–19 amplifiable molecules per reaction for mutation load analysis, or 12–84 amplifiable molecules per reaction for analysis of large expansions. The number of molecules per reaction was calculated according to the Poisson distribution, as previously described [46], [48]. The repeat tract was amplified using primers 147–For and 602-Rev [6]. Products were detected by Southern blot analysis with an end-labeled (TTC)_11_ probe. The sizes of the progenitor alleles were determined by conventional PCR, and mutations were defined as length changes of >5% from the progenitor. Mutation load was calculated as 100× (total number of mutations)/(total molecules analyzed). As we have previously described, the YG8 mice carry multiple copies of the transgene, seen as multiple progenitor/constitutional allele lengths [6] Therefore, expansions and contractions were conservatively defined as products that measured >5% above the longest or below the shortest constitutional allele, respectively. Mutations that measured between the top and bottom progenitors were classified as intermediate changes, since they could have resulted from expansion of a shorter progenitor or contraction of a larger progenitor. The size of an expansion was calculated as the percent increase in length above the longest progenitor allele. Importantly, all comparisons examined instability in the same tissue from wild-type vs. knockout littermates of similar age, or from young vs. old mice from the same line and genotype. In particular, the different number of progenitor alleles and different progenitor allele lengths of the YG8-*Pms2^+/+^* and YG8-*Pms2^−/−^* lines vs. other lines would confound comparisons between these and other lines. Since only the bands that were longer than the longest progenitor allele were considered expansions, the presence of more progenitor alleles would reduce the apparent expansion load. Therefore, comparisons could not be made between different lines (e.g., YG8-*Msh2^+/+^* vs. YG8-*Pms2^+/+^*).

### Statistical analysis

Frequencies were compared by Chi-square test, and medians were compared by Mann-Whitney U test. *P*<0.05 was considered significant.

## Results

### Deficiency of MutSα reduces somatic instability and prevalence of expansions of the expanded (GAA·TTC)_n_ sequence in neuronal tissues

**Figure 1:**
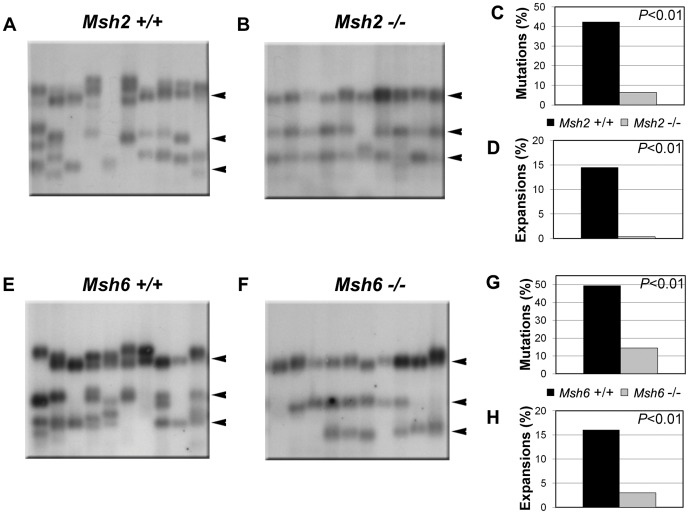
Absence of MutSα reduces somatic mutation load of the (GAA·TTC)_n_ sequence. Panels A, B, E, and F show representative autoradiographs from SP-PCR analysis of DNA extracted from (A) *Msh2^+/+^*, (B) *Msh2^−/−^*, (E) *Msh6^+/+^*, and (F) *Msh6^−/−^* cerebellum. Progenitor allele lengths determined by conventional PCR are 192, 130, and 89 repeats for *Msh2^+/+^*; 188, 128, and 88 repeats for *Msh2^−/−^*; 186, 133, and 98 repeats for *Msh6^+/+^*; and 187, 129, and 90 repeats for *Msh6^−/−^*, as indicated by arrowheads at the right of each panel. Results from the total analysis of 150 to 282 molecules per genotype are quantified in C and D for *Msh2*, and G and H for *Msh6*.

We previously described tissue-specific, age-dependent somatic instability in the YG8 transgenic mouse model carrying (GAA·TTC)_190_ and (GAA·TTC)_82_ sequences in the context of the human *FXN* locus [42]. Mice deficient in individual MMR proteins were crossed onto the background of the YG8 transgenic mouse to generate models for studying the effects of MMR on instability of the expanded (GAA·TTC)_n_ sequence. Small-pool PCR was used to analyze instability in neuronal and non-neuronal tissues in order to detect whether instability increased or decreased in the absence of individual MMR proteins. Cerebellum, cerebrum, and DRG were selected as representative neuronal tissues, and heart and kidney were selected as non-neuronal tissues. Products with repeat lengths that varied in length by more than 5% from the progenitors were considered mutations (see Materials and Methods). Multiple progenitor alleles were generated during the breeding process (e.g., note the three progenitor alleles for Msh2 and Msh6 mice, indicated by arrowheads next to each gel in Figure 1). Any mutation falling between progenitor alleles could have resulted from expansion of a shorter progenitor or contraction of a longer progenitor; such mutations could not be definitively described as either expansions or contractions. Therefore, expansions were conservatively defined as mutations that were larger than the longest progenitor allele, and contractions were similarly defined as mutations that were smaller than the shortest progenitor allele. Mutations that fell between the longest and shortest progenitors were not classified as expansions or contractions, but they were included in the calculation of the total mutation load.

Consistent with our previously published results [6], the prevalence of contractions in all YG8 transgenic mice was much lower than the prevalence of expansions, with many tissues showing no contractions (Tables 1 and 2). The absence of Msh2, Msh6, or Pms2 did not increase the contraction load, indicating that these MMR proteins do not suppress somatic contractions of the (GAA·TTC)_n_ sequence. We could not conclusively determine whether the absence of Msh2 might result in a further decrease in somatic contractions of the (GAA·TTC)_n_ sequence, although any such decrease is unlikely to be biologically relevant given the very low levels of somatic contraction in all tissues analyzed. Based on these data, we conclude that MMR does not affect somatic contractions of the (GAA·TTC)_n_ sequence. Finally, because of the generally low prevalence of contractions, it is quite possible that the unclassified mutations in our analyses are likely the result of expansions from smaller progenitor alleles rather than contractions from larger progenitor alleles.

**Table 1:** Effect of Msh2 and Msh6 on instability in somatic tissues.

	Molecules Analyzed	Expansions∧	Expansion Load, % (*P*)*	Contractions∧	Contraction Load, % (*P*)*	Total Mutations∧	Mutation Load, % (*P*)*
***Msh2 +/+*** ** (10 month)**							
Cerebellum	166	24	14.5	1	0.6	70	42.2
Cerebrum	416	9	2.2	2	0.5	21	5.0
Dorsal Root Ganglia	254	1	0.4	1	0.4	11	4.3
Heart	201	0	0	1	3.7	4	2.0
***Msh2 −/−*** ** (10 month)**							
Cerebellum	282	1	0.4 (<0.01)	5	1.8	18	6.4 (<0.01)
Cerebrum	160	0	0	0	0	2	1.2 (<0.05)
Dorsal Root Ganglia	306	2	0.7	2	0.7	9	2.9
Heart	123	2	1.6	1	0.8	3	2.4
***Msh6 +/+*** ** (11 month)**							
Cerebellum	150	24	16.0	13	8.7	74	49.3
***Msh6 −/−*** ** (12 month)**							
Cerebellum	201	6	3.0 (<0.01)	8	4.0	29	14.4 (<0.01)

* *P* values indicate statistical significance between +/+ and −/−. *P* values are not indicated when not significant.

∧ Because multiple progenitor alleles were present, changes between progenitors could not be definitively identified as contractions or expansions. Contractions and expansions were conservatively defined as molecules that were at least 5% shorter than the smallest progenitor or 5% longer than the largest progenitor, respectively. Contractions, expansions, and changes between progenitors were included in “Total Mutations”.

**Table 2:** Effect of Pms2 on instability in somatic tissues.

	Molecules Analyzed	Expansions∧	Expansion Load, % (*P*)*	Contractions∧	Contraction Load, % (*P*)*	Total Mutations∧	Mutation Load, % (*P*)*
***Pms2 +/+*** ** (12 month)**							
Cerebellum	405	14	3.5	13	3.2	94	23.2
Cerebrum	581	27	4.6	4	0.7	89	15.3
Dorsal Root Ganglia	616	0	0	3	0.5	13	2.1
Heart	117	0	0	1	0.9	2	1.7
Kidney	569	1	0.2	0	0	1	0.2
***Pms2 −/−*** ** (12 month)**							
Cerebellum	451	31	6.9 (<0.05)	8	1.8	107	23.7
Cerebrum	543	49	9.0 (<0.01)	2	0.4	122	22.5 (<0.01)
Dorsal Root Ganglia	666	5	0.8 (<0.05)	0	0	13	2.0
Heart	263	0	0	0	0	5	1.9
Kidney	364	1	0.3	0	0	1	0.3

* *P* values indicate statistical significance between +/+ and −/−. *P* values are not indicated when not significant.

∧ Because multiple progenitor alleles were present, changes between progenitors could not be definitively identified as contractions or expansions. Contractions and expansions were conservatively defined as molecules that were at least 5% shorter than the smallest progenitor or 5% longer than the largest progenitor, respectively. Contractions, expansions, and changes between progenitors were included in “Total Mutations”.

The results of small-pool PCR analysis of the tissues from 10-month old YG8-*Msh2^+/+^* and YG8-*Msh2^−/−^* littermates are shown in Figure 1 (A–D) and summarized in Table 1. In contrast, Msh2 deficiency resulted in a significant and substantial decrease in overall mutation load as well as expansion load in the cerebellum and a significant decrease in overall mutation load in cerebrum. In the heart and DRG, there were virtually no expansions in either genotype, and the absence of Msh2 did not further reduce the already low mutation load (*P*>0.05 in YG8-*Msh2^+/+^* vs. YG8-*Msh2^−/−^*). Because Msh2 is required for initiation of MMR, these results suggest that an active MMR system may be required for somatic expansion of the (GAA·TTC)_n_ sequence.

To further investigate the role of the MMR system in promoting instability of the expanded (GAA·TTC)_n_ sequence, we analyzed somatic instability in cerebellum of 11- to 12-month old YG8-*Msh6^+/+^* and YG8-*Msh6^−/−^* mice. The mutation load was also significantly lower in the YG8-*Msh6^−/−^* versus YG8-*Msh6^+/+^* littermates (Figure 1E–H and Table 1). Together these results indicate that somatic expansion of the expanded (GAA·TTC)_n_ sequence, especially in cerebellum and cerebrum, is dependent on MutSα.

### Pms2 deficiency increases the frequency and magnitude of (GAA·TTC)_n_ expansions in neuronal tissues

**Figure 2:**
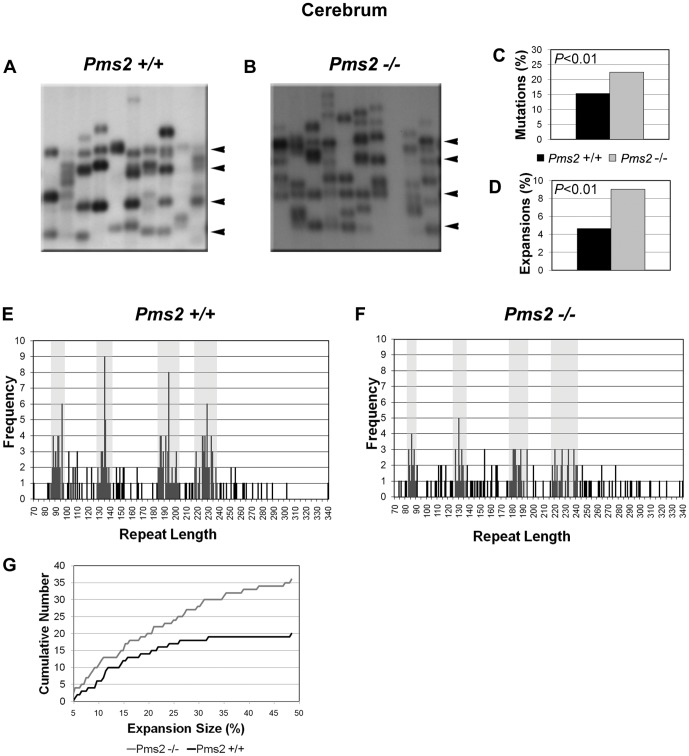
Absence of Pms2 increases somatic expansion load of the (GAA·TTC)_n_ sequence in cerebrum. Analysis of instability in cerebrum of *Pms2^+/+^* and *Pms2^−/−^* mice. Representative autoradiographs from (A) *Pms2^+/+^* and (B) *Pms2^−/−^* cerebrum are shown. Progenitor allele lengths of 229, 194, 135, and 94 repeats for *Pms2^+/+^* and 229, 185, 130, and 86 repeats for *Pms2^−/−^* are indicated by arrowheads at the right of each panel. Mutation load and expansion load, quantified from the analysis of ∼550 molecules per genotype, are shown in panels C and D, respectively. Panels E and F indicate the distribution of repeat lengths for all SP-PCR products of (E) *Pms2^+/+^* and (F) *Pms2^−/−^*. These graphs are therefore an exact, combined representation of all blots analyzed for *Pms2^+/+^* and *Pms2^−/−^* cerebrum. The number of products measured at each repeat length is on the Y-axis. Note that the actual frequency of products at the progenitor lengths may be slightly higher than the counted frequency, because a single band may represent PCR products amplified from multiple progenitor molecules. The gray bands represent the progenitor allele lengths +/−5%. Lines within the gray bands represent unchanged bands. Lines outside of these ranges represent mutations. Those to the right of the rightmost band represent expansions, those to the left of the leftmost band represent contractions, and those in the middle represent unclassified mutations. Panel G shows the cumulative number of products observed with increasing expansion size. Expansion size was calculated as the percent increase in repeat length above the largest progenitor. An incremental increase is seen in *Pms2^−/−^* versus *Pms2^+/+^* mice at all expansion sizes, but the increase in magnitude of the difference at higher expansion sizes points to the role of Pms2 in preferentially suppressing large expansions in the cerebrum.

**Figure 3:**
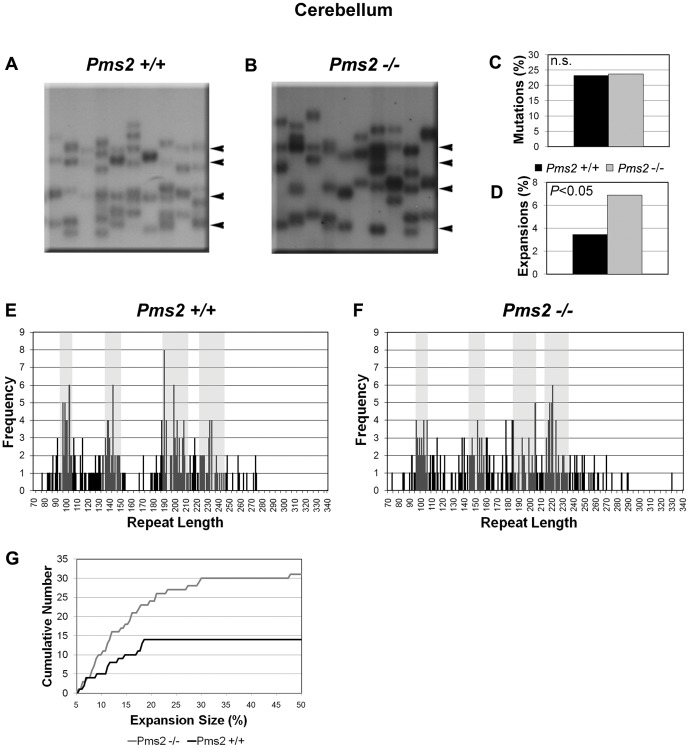
Absence of Pms2 increases somatic expansion load of the (GAA·TTC)_n_ sequence in cerebellum. Analysis of instability in cerebellum of *Pms2^+/+^* and *Pms2^−/−^* mice. Progenitor allele lengths of 232, 199, 143, and 99 repeats for (A) *Pms2^+/+^*, and 224, 195, 152, and 101 repeats for (B) *Pms2^−/−^* are indicated by arrowheads. Mutation load and expansion load, calculated from analysis of ∼400 molecules per genotype, are shown in C and D, respectively. Panels E and F indicate the distribution of repeat lengths for all SP-PCR products of (E) *Pms2^+/+^* and (F) *Pms2^−/−^*. These graphs are therefore an exact, combined representation of all blots analyzed for *Pms2^+/+^* and *Pms2^−/−^* cerebrum. The number of products measured at each repeat length is on the Y-axis. Note that the actual frequency of products at the progenitor lengths may be slightly higher than the counted frequency, because a single band may represent PCR products amplified from multiple progenitor molecules. The gray bars represent the progenitor allele lengths +/−5%. Lines within the gray bands represent unchanged bands. Lines outside of these ranges represent mutations. Those to the right of the rightmost band represent expansions, those to the left of the leftmost band represent contractions, and those in the middle represent unclassified mutations. Products outside of these ranges were counted as mutations. Panel G shows the cumulative number of products observed with increasing expansion size. Expansion size was calculated as the percent increase in repeat length above the largest progenitor. An incremental increase is seen in *Pms2^−/−^* versus *Pms2^+/+^* mice at all expansion sizes, but the increase in magnitude of the difference at higher expansion sizes points to the role of Pms2 in preferentially suppressing large expansions in the cerebellum.

**Figure 4:**
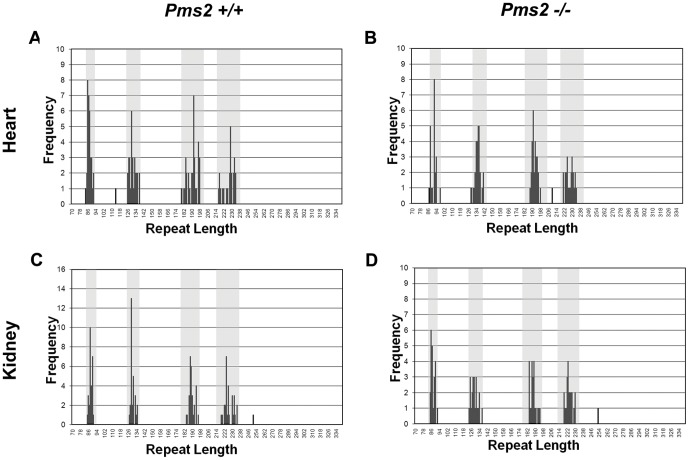
Absence of Pms2 does not affect somatic instability of the (GAA·TTC)_n_ sequence in non-neuronal tissues. Panels show the distribution of repeat lengths measured in (A,B) heart and (C,D) kidney from (A,C) *Pms2^+/+^* and (B,D) *Pms2^−/−^* littermates. The number of products measured at each repeat length is on the Y-axis. Gray bars represent the length of the progenitor alleles, +/−5%. Products that fell outside of these ranges were considered mutations.

Small-pool PCR analysis of somatic instability in tissues from the 12-month old YG8-*Pms2* ^+/+^ and YG8-*Pms2* ^−/−^ mice showed that the absence of Pms2 did not significantly change the mutation load in cerebellum, DRG, heart, or kidney, and only slightly increased the mutation load in the cerebrum. However, Pms2 deficiency significantly increased the prevalence of expansions in cerebrum (Figure 2, Table 2), cerebellum (Figure 3, Table 2) and DRG (Table 2), but not in heart or kidney (Figure 4, Table 2). It is interesting that the expansion frequency in the YG8-*Pms2* ^+/+^ tissues is much lower than that in the YG8-*Msh2^+/+^* and YG8-*Msh6^+/+^* tissues. This observation raises the possibility that the increased expansion load observed in YG8-*Pms2^−/−^* tissues could be an artifact of the low level of expansions in the wild-type. However, we observed significant increases in expansion load in both young and old YG8-*Pms2^−/−^* mice, and these data are consistent with recently published results showing that the loss of Pms2 specifically promotes expansions of repeat sequences, including during intergenerational transmission of the (GAA·TTC)_n_ sequence [47] (see Discussion). Therefore, we conclude that Pms2 may specifically prevent expansions of the (GAA·TTC)_n_ sequence in neuronal tissues.

Furthermore, Pms2 deficiency in the cerebrum significantly increased the prevalence of large expansions, defined as those >10% longer than the longest progenitor allele (Figure 2; 9.0% of amplifiable molecules in YG8-*Pms2^−/−^* vs. 3.4% in YG8-*Pms2^+/+^*; *P*<0.05). In addition, while Pms2 deficiency did not affect the prevalence of large expansions in other tissues, we did observe that expansions exceeding the longest progenitor allele length by >20% occurred only in the cerebellum (n = 7) and DRG (n = 1) of the Pms2 knockout, and not in the wild-type.

**Figure 5:**
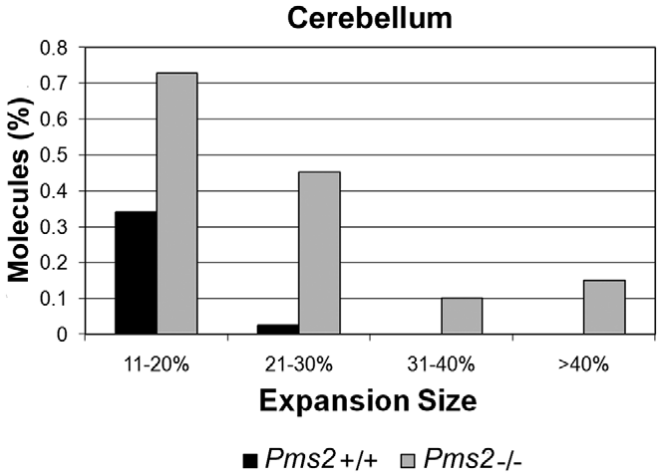
Absence of Pms2 results in larger expansions of the (GAA·TTC)_n_ sequence in cerebellum. SP-PCR was performed using approximately 80 molecules per reaction to detect rare large expansions. Expansion sizes were calculated as the percent increase, of number of repeats, from the longest progenitor. Expansions of at least 10% were considered large expansions. The size distribution of large expansions was significantly different between *Pms2^+/+^* and *Pms2^−/−^* cerebellum (*P*<0.05).

To further investigate the influence of Pms2 deficiency on the prevalence of large expansions in the cerebellum and DRG, we performed additional small-pool PCR experiments using ∼80 amplifiable molecules per reaction to facilitate the detection of rare large expansions. Analysis of >8,000 amplifiable molecules from the cerebellum and >1,500 from the DRG revealed a significant increase in the prevalence of large expansions in the absence of Pms2 (Table 3). The absence of Pms2 also significantly changed the size distribution of expansions in the cerebellum (Figure 5; *P*<0.05). In the absence of Pms2, we observed a median expansion size of 20.2% (*P*<0.005 compared to wild-type), including expansions that were >40% longer than the longest progenitor allele; in contrast, in the presence of Pms2, the median expansion size was 14.8%, with no expansions of >40% and very few expansions of >20%. Together, these data indicate that Pms2 suppresses large expansions of the (GAA·TTC)_n_ sequence in neuronal tissues.

**Table 3:** Effect of Pms2 on large expansions in cerebellum and DRG.

	Molecules Analyzed	Large Expansions	Large Expansion Load, % (*P*)*
***Pms2 +/+*** ** (12 month)**			
Cerebellum	4399	32	0.7
Dorsal Root Ganglia	780	1	0.1
***Pms2 −/−*** ** (12 month)**			
Cerebellum	3985	114	2.9 (<0.001)
Dorsal Root Ganglia	876	10	1.1 (<0.05)
***Pms2 +/+*** ** (5 month)**			
Cerebellum	624	0	0
***Pms2 −/−*** ** (5 month)**			
Cerebellum	907	6	0.7 (<0.05)

* *P* values indicate statistical significance between +/+ and −/−.

*P* values are not indicated when not significant.

Expansions of the (GAA·TTC)_n_ sequence have been shown to accumulate with age, both in humans [5], [49] and in YG8 mice [6]. Therefore, to determine if Pms2 influenced the age-dependent accumulation of expansions, we analyzed the prevalence of large expansions in the cerebellum of 5-month old YG8-*Pms2^+/+^* and YG8-*Pms2^−/−^* mice (Table 3). Rare large expansions were already present at 5 months of age in the knockout, but these were not observed in the wild-type. The prevalence of large expansions was significantly higher at 12 months of age (Table 3), suggesting that Pms2 deficiency also influences the age-dependent rate of accumulation of large expansions of the (GAA·TTC)_n_ sequence in the cerebellum.

## Discussion

Consistent with previous studies on somatic instability of trinucleotide repeats [7], [18]–[22], our data also show that mammalian MutS homologs are required for further somatic expansion of the expanded (GAA·TTC)_n_ sequence in transgenic mice. This observation is also consistent with the observation that expansion of the (GAA·TTC)_n_ sequence in induced pluripotent stem cells (iPSCs) derived from fibroblasts of Friedreich ataxia patients was dependent on Msh2 [50]. Our analysis did not directly assess the role of MutSβ in somatic instability, since the effects of Msh3 were not determined. Our data specifically point to a role for MutSα in somatic expansions, but this does not exclude the possibility that MutSβ participates in somatic instability of the expanded (GAA·TTC)_n_ sequence.

Mismatch repair proteins may act on a lesion or secondary structure of the (GAA·TTC)_n_ sequence that predominantly occurs in neuronal tissues, since instability in non-neuronal tissues was not affected by Msh2, Msh6, or Pms2. Triplexes, R-loops and/or DNA double strand breaks (DSBs) are likely candidates. The (GAA·TTC)_n_ sequence is known to form Y·R·Y triplexes under physiologically relevant conditions [40]. Triplex structures have been shown to promote replication stalling [51], which is known to promote DSB formation [52], and evidence suggests that recombinases may preferentially target expanded (GAA·TTC)_n_ sequences in a Y·R·Y formation [53]. Similarly, R-loops, in which the nascent transcript forms a duplex with the transcriptional template strand, are mutagenic structures that can result in DSBs and recombination via TAR (transcription associated recombination) [54]. R-loops do not normally form during transcription because the nascent transcript is prevented from interacting with the template DNA. However, R-loop formation is known to occur when the (GAA·TTC)_n_ sequence is transcribed [55], and multiple observations suggest that R-loops may be involved in the expansion process. First, with increasing or decreasing transcription of the (GAA·TTC)_n_ sequence in cultured human cells, somatic instability and expansions also increase or decrease, respectively [56], [57]. In addition, data from cultured primate cells implicate a direct role for the GAA-containing transcript in causing expansions of the (GAA·TTC)_n_ sequence [58]. Therefore, factors that regulate somatic expansion of the (GAA·TTC)_n_ sequence could do so by influencing the formation and/or resolution of R-loop formation

Several factors promote R-loop formation, such as defective biogenesis of messenger ribonucleoprotein (mRNP) particles, deficiency of proteins responsible for splicing and/or nuclear export of transcripts, and sequence composition of the non-template strand [54]. For instance, human cells deficient in the ASF/SF2 splicing factor are prone to R-loop formation and DSBs [59]. In addition, both transcription and G-rich sequences, which adopt a G-quartet structure on the non-template strand, facilitate R-loop formation in the switch regions of immunoglobulin (Ig) genes, a requirement for intrachromosomal class switch recombination during antibody isotype switching in mature B cells [60]. The R-loop structure predicted for the transcribed (GAA·TTC)_n_ sequence involves the nascent GAA-containing transcript forming an R-loop structure with the TTC-containing template strand, which would result in a segment of single-stranded non-template strand [55]. This GAA-containing stand would be expected to adopt one or more metastable secondary structures, as the single-stranded GAA sequence is known to do [61], and the very low affinity of polypurine sequences for the eukaryotic single-strand binding protein (replication protein A) [62], would be expected to exacerbate secondary structure formation. In this regard, the R-loops formed during transcription of the (GAA·TTC)_n_ sequence would be similar to those promoted by the G-rich sequences in Ig class switch recombination. In addition, like the R-loops formed in Ig genes, it is possible that one or more of the abnormal structures of the non-template GAA strand could be recognized by MutS homologs, leading to error prone repair. This model is consistent with the observation made by Ku and colleagues, who demonstrated that Msh2 binds in or near the expanded (GAA·TTC)_n_ sequence *in vivo* in iPSCs derived from Friedreich ataxia patients [50].

If the R-loop or triplex structures results in a DSB, as in Ig class switch recombination and in cells deficient in ASF/SF2 splicing factor, then expansions of the (GAA·TTC)_n_ sequence could occur via MMR proteins contributing to error-prone DSB repair. DSB repair, via both the non-homologous end-joining and homologous recombination pathways, is known to occur in neurons and the postnatal brain [63], [64]. Indeed, given the extensive complementarity across the expanded (GAA·TTC)_n_ tract, and its propensity to adopt secondary structures, DSB repair within the expanded (GAA·TTC)_n_ sequence is known to be highly error-prone, with misalignments contributing to repeat instability [65]. It is also noteworthy that the DNA breaks during class switch recombination in B cells actually occur in the G1 phase and are dependent upon MMR [66]. Thus, MutS-initiated error prone repair of secondary structures or DSBs could explain the reduced levels of somatic expansions observed in the mice deficient in MutS homologs.

As part of the MutLα complex, Pms2 links mismatch recognition by MutS homologs to the downstream steps in MMR. Therefore, if the canonical MMR pathway affects somatic instability of (GAA·TTC)_n_ sequences, Pms2 would be expected to have an effect on repeat instability similar to that of the MutS homologs. Indeed, the absence of Pms2 reduced the rate of expansion of the (CAG·CTG)_n_ sequence in transgenic mice by ∼50% [24]. Paradoxically, our results indicate that the absence of Pms2 actually increases the prevalence of large expansions of the (GAA·TTC)_n_ sequence in neuronal tissues, which contrasts with the dramatic reduction of somatic expansions caused by the absence of MutS homologs. The exact reason for this difference is unclear; however, it is noteworthy that Pms2 has also been shown to suppress expansions of other non-hairpin-forming microsatellite repeats, albeit in disparate assays that involved shorter tracts. For instance, in Pms2-null human cell lines, a plasmid-based assay showed a higher percentage of length mutations involving small expansions rather than contractions of the (TTCC·GGAA)_9_ sequence [67]. Similarly, at three different mouse loci containing (CA)_n_ repeats, the absence of Pms2 produced instability that was biased toward expansions in somatic tissues, although these expansions only consisted of small gains [68]. It is interesting to note that the (TTCC·GGAA)_n_ sequence forms a polypurine·polypyrimidine tract similar to the (GAA·TTC)_n_ sequence whereas the (CA)_n_ repeat does not. Indeed, intergenerational transmission of the expanded (GAA·TTC)_n_ sequence by Pms2 null mice led to expansions in the offspring in the majority of transmissions, and this skewing towards expansions was not seen in mice deficient in other MMR proteins [47]. Taken together, these data tend to support a broader role for Pms2 in the prevention of expansion mutations involving a variety of microsatellite repeats, and possibly independent of its role in the MMR pathway.

**Figure 6:**
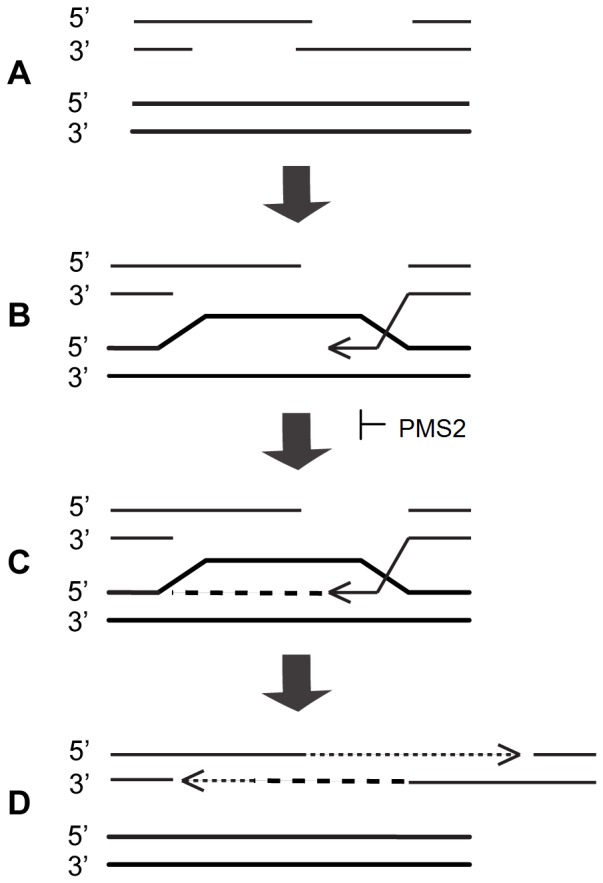
Formation of large expansions due to the absence of Pms2 during double strand break repair. (A) Repair of a double strand break within the repeat sequence begins with 5′ to 3′ resection of the free ends in the broken molecule (thin solid line). (B) The unpaired end of a strand in the broken molecule anneals to a complementary sequence in the template molecule (thick solid line). (C) In the absence of Pms2, DNA synthesis extends the invading strand (thin arrow). In the presence of Pms2, extension is inhibited. The break may be repaired by non-homologous end joining. (D) Erroneous alignment of the newly synthesized DNA (thick dashed line) during reannealing leaves a gap that is filled by another round of synthesis (thin dashed line), resulting in an expansion. The template molecule remains unchanged.

How Pms2 prevents the accumulation of large expansions in neuronal tissues remains unclear; however, it is plausible that this may occur via its ability to act independently of Msh2 in suppressing homologous recombination [69]. It is conceivable that the deficiency of Pms2, and thereby its ability to suppress homologous recombination, could result in large expansions of the (GAA·TTC)_n_ sequence via skewing of the repair mechanism employed for DSB repair (Figure 6). This model would be consistent with the differential effects of Pms2 and MutSα deficiency on the expanded (GAA·TTC)_n_ sequence we observed.

It is unclear why the accumulation of mutations varies substantially among different regions of the nervous system. Potential contributing factors include the distinct variability of cell types composing various regions of the nervous system, differential prevalence of triplexes, R-loops and DSBs, and differential activity and fidelity of DNA repair mechanisms. Indeed, DNA repair activity varies between different regions of the brain [64]. In addition, cellular DSBs, which are usually efficiently repaired in somatic cells to permit cell survival [70], are known to accumulate in postmitotic neurons in an age-dependent manner [71]. These changes could thus allow for the age-dependent accumulation of expansions in specific components of the nervous system. Furthermore, the expanded (GAA·TTC)_n_ sequence became more unstable during intergenerational transmission [47], as opposed to the enhanced stability we observed in somatic tissues of Msh2 and Msh6 null mice. This, and the age-dependent accumulation of mutations in somatic tissues, suggests that the mechanism of expansion and contraction of the (GAA·TTC)_n_ sequence are different in somatic and germ cells.

The progressive expansion of the (GAA·TTC)_n_ sequence in the *FXN* gene in DRG and cerebellum of Friedreich ataxia patients may play a role in influencing the tissue-specific pathogenesis of this disease [5]. Furthermore, the progressive expansion of relatively short alleles within the expanded range may influence the development of mild, late-onset Friedreich ataxia [72]. Therefore, the finding that Pms2 prevents large expansions of the (GAA·TTC)_n_ sequences in neuronal tissues is particularly exciting. Given this unique role, it is tempting to speculate that Pms2 may serve as a genetic modifier of the disease phenotype in Friedreich ataxia.
